# Records from Port Sanitary Authority of Liverpool

**Published:** 1919-10-04

**Authors:** 


### PORTS IN RELATION TO DISEASE.

### Records from Port Sanitary Authority of Liverpool.

A very exhaustive report for the year 1918 on the work of the Liverpool Port Sanitary Authority is provided by the Medical Officer of Health, Professor E. W. Hope, and although it is impossible here to deal with more than a few of the outstanding details, these are of peculiar interest in view of the renewed communications with certain foreign areas which were relatively or completely isolated during the war period. Throughout the author emphasises the urgent necessity of full international understanding and co-operation between port medical authorities, as holding not only one of the most important safeguarding keys to the health of man, but having also intimate relationship to the world's commerce, and we fully agree with his urging that it should be one of the first duties of the new Ministry of Health to set these matters to rights. We understand that the subject is already receiving considerable attention in official circles and wish every success to a very necessary organisation.

### Incidence of Infectious Disease.

The year under review showed a very marked increase in the amount of infectious illness occurring in Liverpool and this was due largely to the arrival of very great numbers of transports carrying American troops, Chinese labour corps, etc. The total number of infectious cases occurring in Liverpool-bound vessels during 1918 was 2,230, as opposed to 104 in 1917. To these must be added 6,353 instances of influenza and 353 of pneumonia during the year, and although these were mainly connected with military forces, the greatest difficulty was experienced in obtaining accommodation for this total with added civilian cases. We can, however, testify from personal experience that the arrangements in Liverpool were, at this period, most excellently carried out and reflect the greatest credit upon those responsible. Apart from influenza, there were in the earlier part of the year a large number of instances of mumps, measles, and other minor diseases, but it is noteworthy that no plague-infected rats were found within the area of the Port of Liverpool and no human cases occurred either in the port or in Liverpool-bound vessels. Smallpox appeared in four separate importations, coincident with a severe outbreak in Portugal and the duration of this voyage being less than the incubation period of the disease, very strict, but fortunately successful, precautionary measures were taken. Educational matters regarding anthrax, were brought to the notice of both workmen and employers with obvious benefit, and the cause of the cases following the use of infected shaving brushes is described as failure efficiently to disinfect a specimen of contaminated goat hair before it was made up into the finished article.

Although cholera cases have not shown alarming increase, Professor Hope draws attention to a definite Western spread during recent times. During the war, in addition to its usual homes in the Middle and Further East, cholera has been widespread in Central and Eastern Europe among the inhabitants of Germany, Austria, and the Balkans. In Russia, Petrograd was again severely attacked, and at the end of the year 1918 there is definite information that Hamburg suffered a serious outbreak. The cases in Palestine will be well remembered and the far-reaching outburst in Bombay in 1918 was of considerable importance as yet further indicating a renewed activity and virulence in practically all the known foci of the disease. With reopening of communications with these areas, a stern tendency to yet further Western spread is to be anticipated, on the experience that this disease follows essentially the trade routes and the lines of travel of the masses.

### Natural Defence against Disease.

Realising, then, the dangers which threaten an island nation with enormous shipping communications, we must appreciate the relatively greater importance to the United Kingdom of our ports as the national barriers of defence against incoming disease. The presence of infection in practically any part of the world whatsoever means its possible introduction sooner or later into a British port, and, of the present situation, as Dr. Hope says, "Absence of a good standard of sanitary administration is usually accompanied by want of appreciation of the degree of risk or the amount of danger associated with infection in the various localities. In other words, maladministration and ignorance are usually associated. For example, no one doubts that the methods adopted when plague is introduced into the average British port rob the incident of any really serious significance so far as dangers to the public health are concerned. The same observation would apply, no doubt, to Japanese or United States, or many other ports, but on the other hand, there are ports in which we know the administration is not effective, and consequently plague at such a port is a menace not only to that port, but to its shipping, and through its shipping to all other ports with which it trades. It is in ports such as these that archaic methods, reminiscent of the old Quarantine Acts, are still in operation, to the great harassing of shipping and obstruction to commerce."

### Needs for the Future.

Gradually must we progress towards the elimination of such potential evils, and the first step is to encourage mutual understanding of both administrative methods and sanitary conditions in the various ports concerned. The utmost advantage would result from the perfection of a system by which the sanitary conditions of all important ports could become common property and common knowledge amongst all of them throughout the world. At present there is far too much suspicion and doubt and "it is unpardonable that commerce should be hampered by avoidable disease, or because one port is regarded as a bogey by another port."

**Ports in Relation to Disease**—(*continued*).

Ideally we need an international standard in this all-important subject and probably the future may see some such development. A good example of its value is provided by one illustration in the case of Liverpool, and, although there are similar arrangements in connection with a number of our other centres, yet Dr. Hope aptly cites it in support of his plea. "The arrival of persons affected with plague at Liverpool has occasionally given rise to delays, costly and vexatious to outward-bound vessels on their arrival at certain ports of the Southern States of America and entails much correspondence and interchange of views with the Consular representatives, Local Government Board, Foreign Office, American authorities, and shipping companies. The view of the Health Department of New York, at which it was apprehended similar restrictions might be imposed, is shown by an extract from the official report, which points out that there may be differences in the problem presented by West Indian and South American ports to those at New York. 'Our commerce with Liverpool,' they state, 'is great in volume and value, and the steamers are huge passenger liners, and the circumstances at Liverpool do not warrant the employment of measures asked for elsewhere, as the result would be to inflict an enormous damage to the commerce of New York not justified by the damage against which it is our duty to guard.' Obviously, if it were possible to enlighten all the ports in the world with which the ports in Great Britain deal, to the same extent as the understanding between the port of Liverpool and the port of New York, it would be to the advantage of all the nations of the world.

